# A proof of principle study using radiopharmaceuticals to quantify and localize container-content interactions in medical syringes

**DOI:** 10.1038/s41598-023-29923-z

**Published:** 2023-02-15

**Authors:** C. Dupire, P. Chennell, B. Pereira, N. Courtiol, S. Buj, R. Cueff, S. Crauste-Manciet, V. Sautou, C. Morgat

**Affiliations:** 1 Pharmaceutical Technology Department, Bordeaux University Hospital, Bordeaux, France; 2 Nuclear Medicine Department, Bordeaux University Hospital, Bordeaux, France; 3 Université Clermont Auvergne, CHU Clermont Ferrand, Clermont Auvergne INP, CNRS, ICCF, 63000 Clermont-Ferrand, France; 4 Department of Clinical Research and Innovation, Clermont-Ferrand University Hospital, Clermont-Ferrand, France; 5 Université Clermont Auvergne, Clermont Auvergne INP, CNRS, ICCF, 63000 Clermont-Ferrand, France; 6 MINT Laboratory UMR INSERM 1066/CNRS 6021, IBS CHU, 4 Rue Larrey, 49933 Angers Cedex 9, France; 7 Pharmacy Department, Angers University Hospital, Angers, France; 8 INCIA, UMR 5287 CNRS, University of Bordeaux, Bordeaux, France

**Keywords:** Imaging techniques, Drug development

## Abstract

The sorption of drugs onto their contents is a known phenomenon that is difficult to analyse precisely. The purpose of this study was to present a non-invasive method for locating and quantifying sorption phenomena using radiopharmaceuticals. Radiopharmaceutical are medicines armed with a radionuclide enabling quantification and imaging using dedicated scanners. The sorption of nine different radiopharmaceuticals on 2- and 3-part syringes was investigated. These syringes were filled with the studied radiopharmaceutical solutions and stored immobile for 3 h. At different times ranging from 0 to 180 min, 10 µL were taken from the syringes and the radioactivity of these samples was determined by a gamma counter. 5 radiopharmaceuticals exhibited no significant sorption at any time point in both 2 and 3-parts syringes, but 4 radiopharmaceuticals exhibited sorption losses varying from 20 to 33% after 3 h contact with 3-part-syringes, but no sorption on 2-part syringes at any time point. [^99m^Tc]Tc-tetrofosmine Single Photon Emission Computed Tomography/Computed Tomography imaging indicated clearly that the interactions were located on the rubber plunger of the 3-part-syringes. The specific nature of radiopharmaceuticals allowed their use as an innovative method to quantify and localize drug sorption phenomena.

## Introduction

Drug sorption (regrouping adsorption and absorption) is a phenomenon by which a material retains compounds, thus potentially modifying the quantity of drug delivered by the device. Adsorption corresponds to the interaction of a drug in solution onto the surface of a material, and is mainly mediated by physisorption. This mechanism is fast and reversible, and can reach a steady state when the adsorption sites are saturated. Adsorption can be followed up by absorption, during which the drug slowly penetrates the material. Despite these interactions being common between medical devices like infusion lines or catheters and lipophilic active pharmaceutical ingredients (API) like for example cyclosporine, tacrolimus, diazepam^1–5^, or with proteins like insulin^6–10^, they remain under-evaluated, especially between medications and syringes, even if recent data does suggest that 3-part syringes can induce losses of diazepam and insulin of more than 20% during a simulated infusion setup^11^. Moreover, very few studies have investigated the impact of these phenomena on the concentrations of radiopharmaceuticals before patient administration. Radiopharmaceuticals are unique medicines either combining a radionuclide for diagnostic (γ and β^+^-emitters) or therapeutic purposes (β^−^ or α-emitters) linked to a carrier molecule or used alone when the radionuclide itself exhibits uptake by the target organ (i.e. iodine isotopes used for imaging and/or treatment of thyroid cancer^12^ or radium isotopes which behave like calcium and are uptaken by bone metastases^13^. Diagnostic radiopharmaceuticals allow non-invasive whole body examination using specific scanners and targeted radioactive treatment^14^. The very unique nature of radiopharmaceuticals also allows them to be monitored by γ-counting, which can be used to quantify their concentration in a solution even at very low doses, as well as allowing them to be detected on inert surfaces, which is something that isn’t easily achievable otherwise.

The main objective of this feasibility study was to investigate if radiopharmaceuticals were at risk of drug loss by sorption during storage in two different types of medical syringes (2-part and 3-part) and quantify the loss if possible. A secondary objective was to identify the parts of the devices causing the interaction.

## Material and methods

### Radiopharmaceuticals

The following radiopharmaceuticals were investigated in this work: [^123^I]I-Ioflupane (GE Healthcare), [^177^Lu]Lu-DOTATATE (Advanced Accelerator Applications, Novartis), [^99m^Tc]Tc-Exametazime-cobalt (GE Healthcare), [^99m^Tc]Tc-Oxidronate sodium (Curium), [^99m^Tc]Tc-Tetrofosmin (GE Healthcare), [^99m^Tc]Tc-Human albumin nanocolloids (GE Healthcare), [^99m^Tc]Tc-Succimer DMSA (Curium), [^99m^Tc]Tc-besilesomab (Curium) and [^223^Ra]Cl_2_ (Bayer). All ^99m^Tc preparations were performed according to the manufacturer's instructions. Radiochemical purity was checked and was greater than 95% for all ^99m^Tc-radiopharmaceuticals. This work was carried out during the stability period of the radiopharmaceuticals.

### Octanol water (LogD_pH5_) and octanol/PBS (logD_pH7.4_) partition coefficients

Octanol/ water and octanol/PBS partition coefficients were determined according to our published methodology^15^. Briefly, 10 kBq of each radiopharmaceutical was added to a centrifuge tube containing 500 μL of 1-octanol and 500 μL of water (pH 5) or phosphate-buffered saline (pH 7.4). After equilibrium, the solution was vigorously stirred for 5 min at room temperature and subsequently centrifuged (4000 rpm, 5 min) to yield two immiscible layers. Aliquots of 100 μL were taken in triplicates, from each layer and the radioactivity (RA) in the samples was determined by a gamma counter (Hidex® and Wizard^2^ automatic gamma counters, 2 min count, ^99m^Tc window: 110–170 keV, ^123^I window: 140–180 keV, ^177^Lu window: 100–140 and 190–245 keV, ^223^Ra window 50–500 keV). Values were decay-corrected. The main nuclear decay data of the radionuclides is summarized in Table 1, and was obtained from NuDat 3.0 (https://www.nndc.bnl.gov/nudat3/) except for ^223^Ra which decays in 6 steps to ^207^Pb. In this specific case, the data from the Summary of Product Characteristics was used.

**Table 1:** Physical properties of the radionuclides used in this work.

	^99m^Tc	^123^I	^177^Lu	^223^Ra
Physical half-life (days)	0.25	0.54	6.64	11.43
Main radioactive emission(s) (energy, intensity)	γ (140.5 keV, 89.0%)	γ (159.0 keV, 83.6%)	β^−^ (496.8 keV, 79.4%) γ (112.9 keV, 6.2%) γ (208.4 keV, 10.4 keV)	α (5.0–7.5 MeV, 95.3%) β^−^ (mean 0.445–0.492 MeV, 3.6%) γ (0.01–1.27 MeV, 1.1%)

### pH measurement

The pH of each radiopharmaceutical solution was measured 10 times by using an indicator strip (VWR-Dosatest® pH test strips 0–14).

### Preparation of the syringes containing the radiopharmaceuticals

Two types of 5 mL syringes were investigated: two-part syringes (2P) (Discardit II 309050, BD Medical®) and three-part syringes (3P) (Medicina IVL05, Medicina France®). Component materials (obtained from the product data sheet) of these syringes are described in Table 2.

**Table 2:**
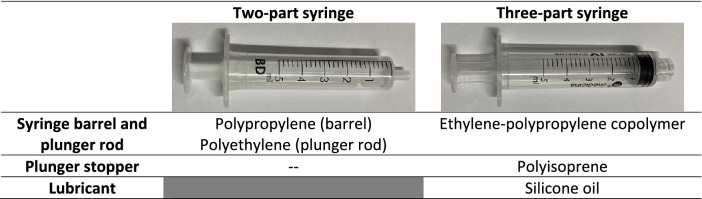
Composition of the tested 2 and 3-part syringes.

For each experiment, the radiopharmaceutical was withdrawn from the stock solution (≤ 0.74 mL) into a syringe (2P or 3P, in triplicates each) and the syringe was then filled up to 5 mL with sodium chloride 0.9% (Proamp®) except for [^123^I]I-Ioflupane which was filled with water for injection (Proamp®) as recommended by the Summary of Product Characteristics. We assumed a homogeneous distribution of the radiopharmaceuticals after filling the syringe with the solvent, as the volume of saline solution sucked up into the syringe (of at least 4.26 mL) was much higher than that of the radiopharmaceutical and that the turbulent flow created by the suction would efficiently mix the volume. In each case, the concentrations (ng/mL) and amounts (ng) of the radiopharmaceuticals was calculated then reported from the manufacturer's data (see Supplemental Table 1).

### Investigation of the radiopharmaceutical sorption

At selected time points (0, 2, 5, 10, 30, 60, 120 and 180 min) after syringe preparation, ten microliters (in triplicate) were carefully withdrawn (using a calibrated pipette) from the syringe and deposited in a 5 mL tube. Additionally, after the last sampling at 180 min, we also investigated the impact of homogenising the solution by 10 inversions of the syringe. The radioactivity in the tubes was determined by a gamma counter as described above. During the waiting period, the syringes were stored at room temperature, protected from light.

For data analysis, 0 min was set as reference. At each time point (T), the percentage of radiopharmaceutical that does not undergo sorption-type interactions with the syringe was determined according to the following equation:

$${\text{No}}\;{\text{sorption }}\left( \% \right) \, = \, \left( {{\text{RA }}\left( {\text{T}} \right) \, /{\text{ RA }}\left( 0 \right)} \right) \, *{1}00)$$

### Contact angle measurements

Contact angle measurements were performed on the polypropylene barrel and on the plunger stopper using the sessile drop method. In brief, a small drop of deionized water (4 μL) was laid down on the surface of the plunger or barrel and analysed with a contact angle measuring system (Krüss Drop Shape Analyzer DSA 14) involving angle measurements in a normal plane. During image analysis, the water contact angle between the baseline of the drop and the tangent at the drop boundary was calculated with the DSA software (Easydrop, version 1.92.1.1, Hamburg, Germany).

### Single photon emission computed tomography/computed tomography (SPECT/CT) imaging

Feasibility of imaging of radiopharmaceutical interactions with the plunger stopper of 3-part syringes was evaluated using SPECT/CT. Two cases were investigated: (i) no significant interactions neither on 2-part syringes nor 3-part syringes using [^99m^Tc]Tc-oxidronate sodium; (ii) significant interaction on 3-part syringes and no interactions on 2-part syringes using [^99m^Tc]Tc-tetrofosmin. Syringes were filled with the radiopharmaceuticals ([^99m^Tc]Tc-oxidronate sodium (127.95 ± 16.04 MBq) or [^99m^Tc]Tc-tetrofosmin (11.93 ± 2.03 MBq) as described above). Two hours after, syringes were emptied and slowly rinsed with 5 mL of a saline solution (NaCl 0.9%) sucked up into the syringe and gently used to rinse the inside of the syringe, then emptied out. SPECT images were performed with a dual head gamma camera (Discovery NM/CT 670, GE Healthcare) equipped with low-energy thin-section collimators in tomographic mode. Camera settings were 256 × 256 acquisition matrix, 10% energy window at 140.5 keV, 30 projections per detector, 60 s per projection. SPECT images were reconstructed using an OSEM algorithm on a 256 × 256 matrix using iterative reconstruction (3 iterations, 10 subsets, Xeleris workstation v3). CT parameters were 120 keV ± 5% for attenuation correction. Images were also corrected for diffusion and resolution recovery. Post-filter parameters were: butterworth, cutting frequence 0.55 and order 10. Experiments were performed twice in triplicates.

### Data and statistical analyses

Values were expressed as mean and standard error of the mean (SEM). The normality was analyzed using a Shapiro–Wilk test. To compare the sorption kinetics between 2 and 3-part syringes of each radiopharmaceutical, random-effects models were performed to take into account longitudinal repeated data. The following fixed effects were evaluated: group (2 and 3-part syringes), time and their interaction. The normality of residuals was analyzed as aforementioned. When appropriate, a logarithmic transformation of sorption was applied. Statistical analyses were conducted using Stata software (version 15, StataCorp, College Station, US) with a two-sided type I error at 5% corrected due to multiple comparisons. More precisely, a Sidak’s type I error correction was applied. Furthermore, sorption was considered clinically significant if the loss was ≥ 10%. A sorption < 10% but statistically significant was considered as no sorption. This hypothesis is in line with the current practice of accepting a ± 10% uncertainty on the activity injected compared to the prescribed activity.

## Results

### Physicochemical properties of the radiopharmaceutical solutions

Physicochemical properties of the radiopharmaceuticals investigated in this study are summarized in Table 3. Molecular weight and charges were obtained from the literature. (Corresponding references are in superscript in Table 3):

**Table 3:** Physicochemical properties of the radiopharmaceuticals investigated in this work. MW = molecular weight.

Radiopharmaceuticals	MW (Da)	pH	Log D (pH 5)	Log D (pH 7.4)	Charge (at the pH value reported in column 3)
[^177^Lu]Lu-DOTATATE	1610^1^	5.20 ± 0.26	− 1.97 ± 0.15	− 1.94 ± 0.01	unknown
[^99m^Tc]Tc-Exametazime	384^2^	7.00 ± 0.24	0.47 ± 0.46	0.75 ± 0.12	0^3^
[^99m^Tc]Tc-Oxidronate	290^4^	5.35 ± 0.24	− 2.16 ± 0.18	− 2.9 ± 0.05	0^5^
[^123^I]I-Ioflupane	427^6^	4.50 ± 0.00	0.15 ± 0.21	0.55 ± 0.00	0^6^
[^223^Ra]Cl_2_	294^7^	7.60 ± 0.39	− 2.89 ± 0.03	− 2.90 ± 0.02	+ 2^8^
[^99m^Tc]Tc-Besilesomab	≈ 150,000^9^	6.95 ± 0.28	− 0.91 ± 0.06	− 1.08 ± 0.05	0^9^
[^99m^Tc]Tc-Tetrofosmin	895^10^	7.85 ± 0.34	0.27 ± 0.23	0.56 ± 0.09	+ 1^10^
[^99m^Tc]Tc-DMSA	456^11^	3.05 ± 0.16	− 0.46 ± 0.32	− 1.55 ± 0.25	− 3^11^
[^99m^Tc]Tc-Nanocolloids	≈ 670,000^12^	6.65 ± 0.41	− 1.55 ± 0.08	− 1.08 ± 0.16	Unknown

### Container content interaction

**Figure 1:**
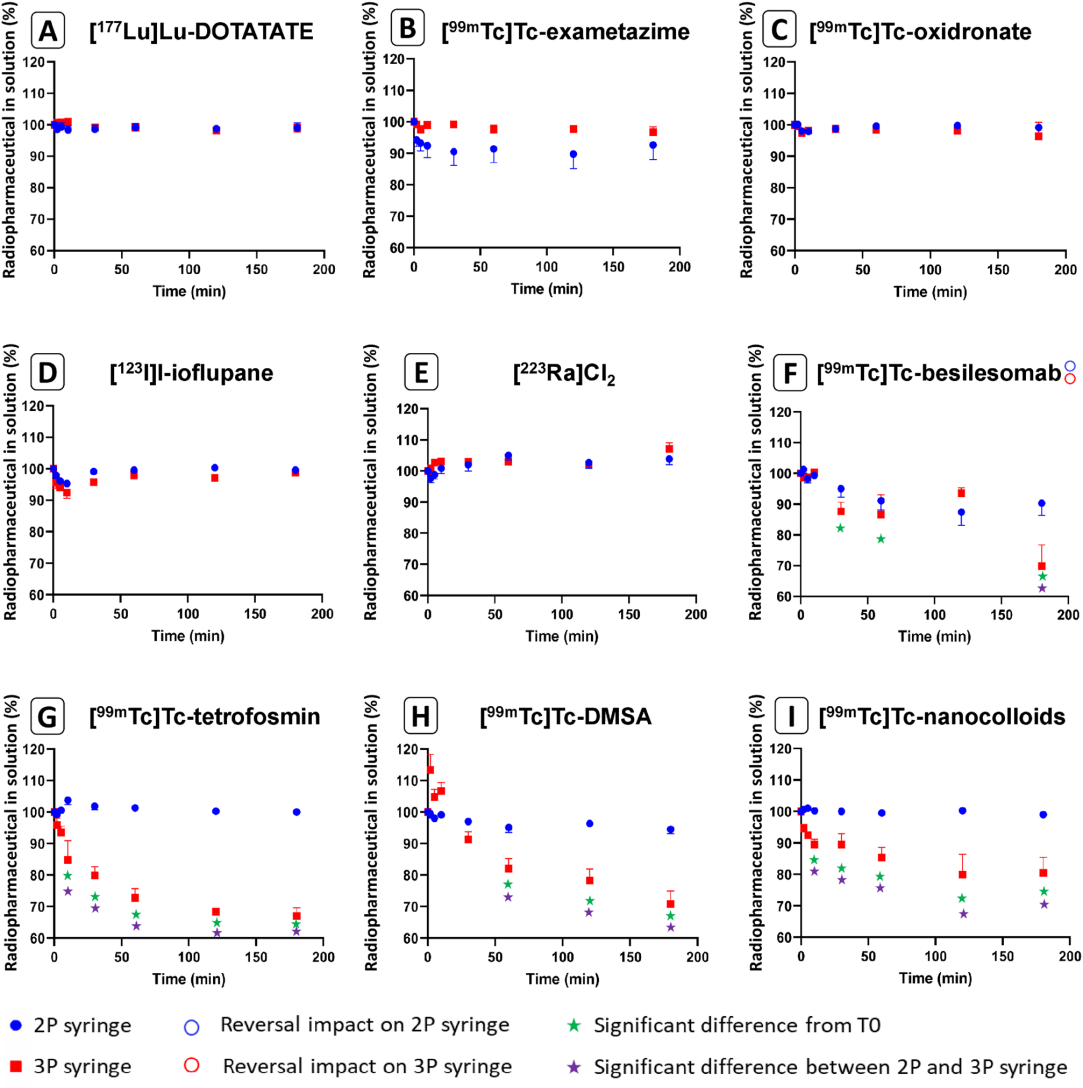
Kinetics of the radioactivity measured from the nine radiopharmaceuticals in 2 and 3-part syringes.

The sorption kinetics of each radiopharmaceutical in 2 and 3-part syringes are depicted in Supplemental Table 2 and summarized in Fig. 1.

[^177^Lu]Lu-DOTATATE; [^99m^Tc]Tc-exametazine, [^99m^Tc]Tc-Oxidronate sodium, [^123^I]I-ioflupane and [^223^Ra]Cl_2_ exhibited no significant sorption at any time point in both 2 and 3-parts syringes (p > 0.05).

[^99m^Tc]Tc-besilesomab, [^99m^Tc]Tc-tetrofosmin, [^99m^Tc]Tc-DMSA and [^99m^Tc]Tc-nanocolloides showed no sorption on 2-part syringes at any time point. On 3-part-syringes, a significant difference was found from 30 min onwards (p ≤ 0.002), for [^99m^Tc]Tc-besilesomab, from 10 min onwards for [^99m^Tc]Tc-tetrofosmin and for [^99m^Tc]Tc-nanocolloids (p < 0.001) and a significant loss was demonstrated from 60 to 180 min for [^99m^Tc]Tc-DMSA (p < 0.001). Additionally, all these radiopharmaceuticals displayed a significantly higher sorption for 3-part syringes compared with 2-part syringes at 180 min. Comparison of sorption between 2-part and 3-part syringes revealed a higher sorption of [^99m^Tc]Tc-besilesomab on 3-parts syringes at 180 min (p < 0.001). Similarly, sorption of [^99m^Tc]Tc-tetrofosmin and [^99m^Tc]Tc-nanocolloids was significantly higher on 3-part syringes compared to 2-part syringes from 10 to 180 min (p < 0.001 except for [^99m^Tc]Tc-nanocolloids at 10 min p = 0.001) and [^99m^Tc]Tc-DMSA showed also higher sorption on 3 part syringes from 60 to 180 min (p = 0.007 at 60 min and p < 0.001 from 120 to 180 min).

At 180 min, each syringe underwent 10 reversals. This reversal was significant only for [^99m^Tc]Tc-besilesomab, which showed lower sorption after reversal on 3-part syringes (p < 0.001). Sorption of all other radiopharmaceutical were not modified by this reversal (p > 0.05).

### Contact angle measurements

Contact angle measurement results are presented in Table 4. The polyethylene plunger rod surface presented a much lower contact angle (75.3°) than the polyisoprene plunger stopper (105.2°), thus indicating that the polyisoprene is much more hydrophobic.

**Table 4:** Contact angle measurements results, expressed in degree (°) as mean ± standard deviation.

	Sample 1	Sample 2	Sample 3	Mean (24 measurements)
2-part syringe (polyethylene plunger rod)	74.4 ± 4.1	76.6 ± 4.1	74.9 ± 3.0	75.3 ± 3.7
3-part syringe (polyisoprene plunger seal)	104.7 ± 2.2	105.4 ± 2.5	105.6 ± 2.4	105.2 ± 2.3

Eight measurements were performed for each sample.

### Single photon emission computed tomography/computed tomography (SPECT/CT) imaging

**Figure 2:**
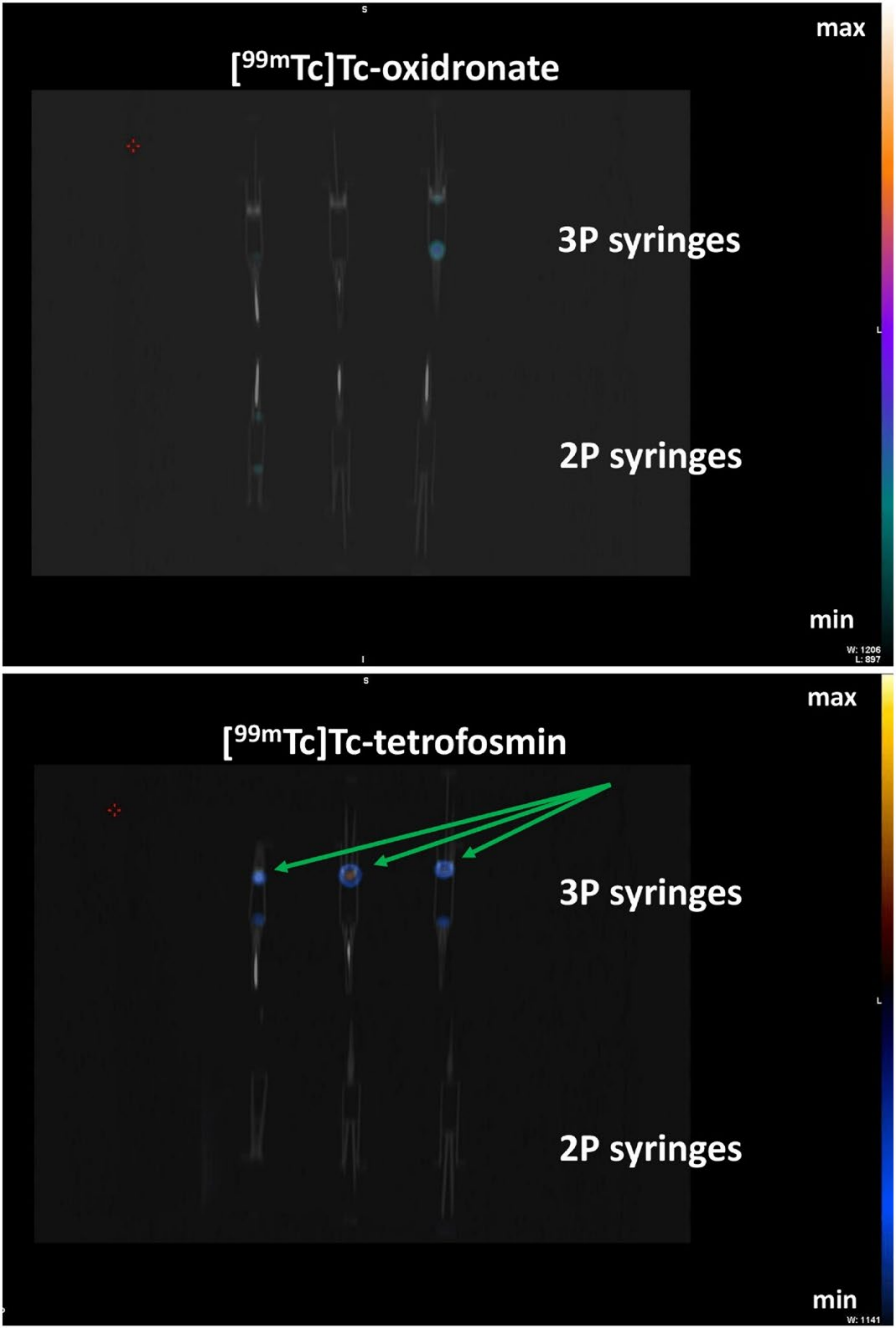
SPECT/CT imaging of [^99m^Tc]Tc-oxidronate and [^99m^Tc]Tc-Tetrofosmin on 2-part and 3-part syringes. Black and white refer to the CT acquisition. Colors refer to radioactive SPECT signal. The green arrows point to the major sites of sorption (plunger stopper).

Owing to the γ-emission of the radiopharmaceuticals investigated, a feasibility study using SPECT/CT imaging was performed to localize the area of sorption. [^99m^Tc]Tc-oxidronate and [^99m^Tc]Tc-tetrofosmin were used. As expected, [^99m^Tc]Tc-oxidronate showed no sorption on SPECT/CT imaging both on 2P and 3P syringes (Fig. 2). The activity seen on a 3P syringe is in fact a residual drop. Interestingly, SPECT/CT imaging of 2P and 3P syringes previously filled with [^99m^Tc]Tc-tetrofosmin showed activity only on the plunger stopper of 3P syringes (Fig. 2). This is the first report of the use of nuclear imaging to localize the area of sorption.

## Discussion

API sorption to medical devices is a relatively old problem, but which unfortunately is still relevant today, thus possibly altering patient care^16^. Usual methods used to investigate drug sorption are aimed at evaluating a loss of API in the medication in contact with the devices, mainly by using high-performance liquid chromatography^17^ and/or mass spectrometry^10^. Unfortunately, these methods do not allow the localization of the API sorption site on the devices, therefore making the identification of the cause more difficult. In this work we used a less investigated class of medicines (radiopharmaceuticals), which enabled us to perform non-invasive quantification and localization of drug sorption during simulated drug storage in 2-part and 3-part syringes. Our main results showed that the 3-part syringes caused drug loss by sorption in 4 out of the 9 radiopharmaceuticals that were tested, and SPECT/CT imaging allowed us to pinpoint the sorption site as being the polyisoprene plunger.

The nine radiopharmaceuticals that were used in this study had different physicochemical properties (in molecular weight and charge notably), thus covering a wide range of medications used in nuclear medicine, including a monoclonal antibody. These drugs can be prepared by the radiopharmacy unit up to a couple of hours before patient administration, and are therefore also at risk of container-content interactions with the devices (syringes) they are stored in. In this proof of principle study, we restricted our study to the two most commonly used types of syringes: 2 pieces and 3 pieces, differing mainly by the presence of a rubber plunger stopper in the 3-part syringes which is absent in the 2-part syringes.

After analysis of the results, two distinct groups of radiopharmaceutical can be easily identified: those exhibiting no significant sorption either on the 2P-syringe, nor on the 3-part syringe ([^177^Lu]Lu-DOTATATE; [^99m^Tc]Tc-exametazine, [^99m^Tc]Tc-Oxidronate sodium and [^123^I]I-ioflupane); and those exhibiting significant sorption on 3-part syringes ([^99m^Tc]Tc-besilosumab, [^99m^Tc]Tc-tetrofosmin, [^99m^Tc]Tc-DMSA and [^99m^Tc]Tc-nanocolloids). For these medications, the losses that were measured ranged from 26 to 41% ([^99m^Tc]Tc-besilosumab and [^99m^Tc]Tc-tetrofosmin, respectively), which is a considerable loss that could induce mis-diagnostic and patient under-dosing. Also, the kinetics of the interaction were very fast, the loss being higher than 10% after 30 min of contact for [^99m^Tc]Tc-besilesomab, [^99m^Tc]Tc-tetrofosmin, and [^99m^Tc]Tc-nanocolloids, and even of 15% for [^99m^Tc]Tc-tetrofosmin after only 10 min. The difference in behavior between types of syringes clearly incriminates the plunger stopper and/or the silicone oil that is added as lubricant^18,19^. This hypothesis is strongly supported by contact angle measurements and by SPECT/CT imaging. The contact angle measurements showed that the contact angle formed on the barrel surface (polyethylene) was lower than that obtained on the rubber plunger (75.3° versus 105.2°, Table 4). The barrel surface therefore possesses a slightly hydrophilic nature, which can seem surprising for polyethylene as the values generally reported are closer to 90°, which sets it a the limit between hydrophobic and hydrophilic materials. This could be explained by the effect of the sterilization method, which has been shown in certain cases to lower the water/polyethylene contact angle, leading therefore to a more hydrophilic surface^20,21^. This decrease of contact angle measurement to around 75 °C has more specifically been reported by Castle et al., during polyethylene sterilization by hydrogen peroxide^20^. In our case the devices were sterilized using ethylene oxide, but it can be hypothesized that the surface effect would be similar. The 105° contact angle measured for the rubber plunger is consistent with the results reported in the literature for this type of surface^22–24^ and is characteristic of hydrophobic surfaces. SPECT/CT imaging allowed us to precisely pinpoint the location of the interaction as being caused by the plunger stopper, as shown in Fig. 2. To our knowledge this is the first published study using this sort of analysis to help identify the causal material of a sorption phenomenon and opens up interesting perspectives for future studies for example of other devices. This finding is also coherent with the Supplementary data provided in some recently published work showing a loss of diazepam ranging from 25% to more than 50% when left in contact with syringe plungers^11^. Additionally, it seems to indicate that polyisoprene is not inert, and caution should be applied when using this material in contact with medications.

Careful analysis of the radiopharmaceuticals presenting drug sorption compared with those not having sorption highlighted molecular weight and/or molecular net charge as parameters that might drive drug sorption. For instance, [^99m^Tc]Tc-besilesomab and [^99m^Tc]Tc-nanocolloids of high molecular weight (but not with a net charge of 0) presented drug sorption. In this case, surface adsorption might be associated with the high molecular weight, as an increased size could lead to the existence of specific chemical moieties that might have an increased affinity for the plunger seal surface. Contrarily, [^99m^Tc]Tc-tetrofosmin and [^99m^Tc]Tc-DMSA, which are APIs of low molecular weight (895 Da and 456 Da, respectively, see Table 3), but with a net charge of + 1 and − 3, also presented significant sorption when in contact with 3-part syringes. Herein, electrostatic interactions might be incriminated. However, [^223^Ra]Cl_2_ possesses a net charge of + 2 and is of low molecular weight (294 Da), but exhibited no sorption. Drug lipophilicity (expressed by the Log D at pH 5 and pH 7.4) didn’t seem to be a parameter with which a correlation with sorption could be found, as out of the 4 radiopharmaceuticals that exhibited important losses by sorption, only one ([^99m^Tc]Tc-Tetrofosmin) had a positive Log D (thus indicating a more lipophilic nature). The two other medications in this study with a positive Log D at pH 5 and 7.4 ([^99m^Tc]Tc-Exametazime and [^123^I]I-Ioflupane) didn’t present any signs of sorption on the 3-part syringes. This is surprising as for non-radiopharmaceuticals drug lipophilicity has often been regarded as one of the main factors driving absorption. It is possible therefore that all the drug loss seen is this work results only from surface adsorption.

This study is the first of its kind using radiopharmaceuticals to quantify and localize sorption mechanisms, and as such was designed as a proof of principle study. Also, the radiopharmaceuticals used are for some of them very innovatives (i.e. [^177^Lu]Lu-DOTATATE and [^223^Ra]Cl_2_) and used only in specialized centers. However, despite it having been conducted with 9 different drugs, it was not possible to draw any definite conclusions concerning the factors that influence sorption. For such conclusions to be made, it would need to be enlarged into a systematic and comparative analysis including more materials, syringes and infusion systems. Also, some clinically used radionuclides with short half-lives [i.e. ^68^ Ga (67.6 min), ^82^Rb (78 s), ^81m^Kr (13 s)] cannot be used for drug sorption kinetics studies, thus limiting the number of radiopharmaceuticals suitable for this kind of investigations. Lastly, the limited availability of some radiopharmaceuticals with unique physicochemical parameters may not allow extensive studies. But despite this, the methodology presented in this work remains promising, and other applications, such as for example using radiopharmaceuticals as diagnostic companions during the drug development process would be recommended to non-invasively assess container-content interaction into/onto the targeted infusion system. Examples reported in the literature accounted for [^195m^Pt]Pt-cisplatin^25^, [^99m^Tc]Tc-antibiotics^26^, [^99m^Tc]Tc-doxorubucin^27^ or [^89^Zr]Zr-trastuzumab^28^ that include narrow therapeutic range APIs could in the future benefit from sorption evaluation as early as possible during their drug development.

The nuclear medicine/radiopharmacy field would also benefit from the results of this study. For instance, radiopharmaceuticals are usually produced (centrally or locally) using automated synthesizers which use plastic and glass materials that can cause loss of product during the process. Therefore, it would be feasible to quantify and localize radiopharmaceutical sorption in order to favor materials with low sorption, thus leading to higher production yields and a better cost-effectiveness of the production. A recent study highlighted that fluorinated tubing, used for the transportation of ^18^F-fluoride from the cyclotron to the synthetizor, caused a significant decrease in molar activity of ^18^F-radiopharmaceutical^29^. Another major application of this study is the receptor saturation that can be achieved when low molar activity of receptor-targeted radiopharmaceuticals are used. This is particularly true in preclinical research as grafted tumors (orthotopic or xenograft) might have lower receptor density than humans and may be more sensitive to changes in molar activity. For example, uptake on imaging of ^68^Ga-radiolabeled Prostate Specific Membrane Antigen (PSMA) inhibitors is dependent on their molar activity^30^ and the therapeutic efficacy of radiolabeled cholecystokinine analogues is limited when receptor saturation is achieved^31^. Finally, this finding also apply to studies in humans as the peptide mass of radiolabeled PSMA inhibitors influences clinical PET imaging in selected prostate cancer patients^32^. Also, high administered peptide amount of [^177^Lu]Lu-HA-DOTATATE (a somatostatin receptor agonist) leaded to lower uptake in neuroendocrine tumors overexpressing somatostatin receptors^33^.

This study suffers from several limitations. As discussed above, the molar activity might influence imaging and therapy in nuclear medicine. This is also true for sorption phenomena. In this work, for some radiopharmaceuticals, similar molar activities have been used and might have saturated the material and therefore may not correctly reflect the behavior of higher molar activities. Moreover, we did not find any association between physicochemical parameters and sorption. This point could be investigated more in-depth in future work. Finally, the SPECT/CT acquisition used to build the image to localize the site of container content interaction was performed using a clinical device with a sub centimeter resolution. Recent advances in multi-pinhole technologies in microSPECT imaging have pushed the resolution down to 100 µm, according to the radionuclide used^34^. Therefore, imaging materials using a µSPECT device would provide a better localization of the site of sorption.

## Conclusion

In this work, we proved, using an innovative analytical method, that radiopharmaceuticals can quantify and localize drug loss due to sorption phenomena with the polyisoprene plunger seal of 3-part syringes, after as little as only a couple of minutes of storage. 3-part syringes should be used with caution are for the preparation of radiopharmaceuticals when sorption data is not available, as even if sorption was proven here with polyisoprene, other plunger stopper materials could also be at risk. Although this method cannot discriminate adsorption from absorption phenomena, it can be systematically used during the development process of a new drug and/or a new material. For this purpose, we encourage the development of companion, radiolabeled drugs.

## Supplementary Information

Supplementary Tables.

## Data Availability

All data are reported in the manuscript and its Supplemental file.
