# Supplementary material for: A proof of principle study using radiopharmaceuticals to quantify and localize container-content interactions in medical syringes
Source: Sci Rep. 2023 Feb 15;13:2721. doi: 10.1038/s41598-023-29923-z (PMC9932089; doi:10.1038/s41598-023-29923-z)
Supplement: Supplementary file 1 — Supplementary Tables. [file 41598_2023_29923_MOESM1_ESM.docx]

**Supplemental information**

**A proof of principle study using radiopharmaceuticals to quantify and localize container-content interactions in medical syringes**

Dupire C ^a,b,1^, Chennell P ^c,1^, Pereira B ^d^, Courtiol N ^a,b^, Buj S ^b^, Cueff R ^e^, Crauste-Manciet S ^f,g^, Sautou V ^c^, Morgat C ^b,h,*^.

^a^ Pharmaceutical Technology Department, Bordeaux University Hospital, Bordeaux, France

^b^ Nuclear Medicine Department, Bordeaux University Hospital, Bordeaux, France

^c^ Université Clermont Auvergne, CHU Clermont Ferrand, Clermont Auvergne INP, CNRS, ICCF, F-63000 Clermont-Ferrand, France

^d^ Department of Clinical Research and Innovation, Clermont-Ferrand University Hospital, Clermont-Ferrand, France

^e^ Université Clermont Auvergne, Clermont Auvergne INP, CNRS, ICCF, F-63000 Clermont-Ferrand, France

^f^ MINT Laboratory UMR INSERM 1066 /CNRS 6021, IBS CHU, 4 rue Larrey 49933 Angers cedex 9 France

^g^ Pharmacy Department, Angers University Hospital, Angers, France

^h^ INCIA, UMR 5287 CNRS, University of Bordeaux, Bordeaux, France

^1^ Equally contribution

***** Corresponding author at: Nuclear Medicine Department, Bordeaux University Hospital, Bordeaux, France. *Email address:* [clement.morgat@chu-bordeaux.fr](mailto:clement.morgat@chu-bordeaux.fr) (C.Morgat)

Authors state no conflict of interest

Supplemental Table 1: Concentrations (ng/mL) and amounts (ng) of the radiopharmaceuticals.

| **Radiopharmaceuticals** | **Syringe type** | **Concentration (nM)** | **Amount (ng)** |
| --- | --- | --- | --- |
| [^177^Lu]Lu-DOTATATE | 2P | 421.49 | 3393.00 |
|  | 2P | 16.86 | 135.72 |
|  | 2P | 15.45 | 124.41 |
|  | 3P | 561.99 | 4524.00 |
|  | 3P | 25.29 | 203.58 |
|  | 3P | 44.96 | 361.92 |
| [^99m^Tc]Tc-exametazime | 2P | 22321.43 | 42857.14 |
|  | 2P | 1004.46 | 1928.57 |
|  | 2P | 2269.35 | 4357.14 |
|  | 3P | 23809.52 | 45714.28 |
|  | 3P | 558.04 | 1071.42 |
|  | 3P | 1376.49 | 2642.86 |
| [^99m^Tc]Tc-oxidronate | 2P | 95172.41 | 138000.00 |
|  | 2P | 79310.34 | 115000.00 |
|  | 2P | 186206.90 | 270000.00 |
|  | 2P | 951.72 | 1380.00 |
|  | 3P | 82758.62 | 120000.00 |
|  | 3P | 255172.41 | 370000.00 |
|  | 3P | 3620.69 | 5250.00 |
|  | 3P | 2758.62 | 4000.00 |
| [^123^I]I-ioflupane | 2P | 0.84 | 18.00 |
|  | 2P | 1.41 | 3.00 |
|  | 2P | 1.73 | 3.70 |
|  | 3P | 3.37 | 7.20 |
|  | 3P | 2.30 | 4.90 |
|  | 3P | 1.45 | 3.10 |
| [^223^Ra]Cl_2_ | 2P | 8.14E-03 | 0.0120 |
|  | 2P | 8.14E-03 | 0.0120 |
|  | 2P | 9.09E-03 | 0.0134 |
|  | 3P | 8.14E-03 | 0.0120 |
|  | 3P | 8.14E-03 | 0.0120 |
|  | 3P | 9.09E-03 | 0.0134 |
| [^99m^Tc]Tc-besilesomab | 2P | 131.11 | 98333.33 |
|  | 2P | 24.44 | 18333.33 |
|  | 2P | 21.11 | 15833.33 |
|  | 3P | 115.56 | 86666.67 |
|  | 3P | 4.44 | 3333.33 |
|  | 3P | 11.11 | 8333.33 |
| [^99m^Tc]Tc-tetrofosmin | 2P | 835.20 | 3737.50 |
|  | 2P | 57.18 | 255.88 |
|  | 2P | 25.70 | 115.00 |
|  | 3P | 1092.18 | 4887.00 |
|  | 3P | 70.67 | 316.25 |
|  | 3P | 308.38 | 1380.00 |
|  | 3P | 83.52 | 373.75 |
| [^99m^Tc]Tc-DMSA | 2P | 24561.40 | 56000.00 |
|  | 2P | 1315.79 | 3000.00 |
|  | 2P | 2631.58 | 6000.00 |
|  | 3P | 29824.56 | 68000.00 |
|  | 3P | 13157.89 | 30000.00 |
|  | 3P | 4912.28 | 11200.00 |
|  | 3P | 2543.86 | 5800.00 |
|  | 3P | 4649.12 | 10600.00 |
| [^99m^Tc]Tc- nanocolloids | 2P | 6.57 | 22000.00 |
|  | 2P | 16.42 | 55000.00 |
|  | 2P | 4.08 | 13666.67 |
|  | 3P | 12.44 | 41666.67 |
|  | 3P | 0.72 | 2400.00 |
|  | 3P | 2.90 | 9700.00 |
|  | 3P | 1.55 | 5200.00 |

Supplemental Table 2 : Sorption kinetics of each radiopharmaceutical in 2 and 3-part syringes. In red: statistically significant variations but considered as being non-clinically significant when including the standard error of the mean. In green: statistically significant variations.

|  |  | **Comparaison to T0 (T0= 100%)** | | | | | | | |
| --- | --- | --- | --- | --- | --- | --- | --- | --- | --- |
| **Radiopharmaceutical** | **Syringe type** | **T2** | **T5** | **T10** | **T30** | **T60** | **T120** | **T180** | **T180+R** |
| **[^177^Lu]Lu-DOTATATE** | 2P | 98.57  ± 1.51 %  *p = 0.282* | 99.38  ± 1.27 %  *p = 0.596* | 98.38  ± 1.32 %  *p = 0.178* | 98.63 ± 1.29 %  *p = 0.254* | 99.17 ± 1.25 %  *p = 0.542* | 98.78  ± 0.62 %  *p = 0.300* | 99.00  ± 1.56 %  *p = 0.405* | 100.40  ± 1.08 %  *p = 0.556* |
|  | 3P | 100.63  ± 1.45 %  *p = 0.655* | 100.67 ± 1.84 %  *p = 0.940* | 101.00  ± 1.93 %  *p = 0.995* | 99.13 ± 0.99 %  *p = 0.346* | 99.13 ± 1.08 %  *p = 0.386* | 98.25  ± 0.65 %  *p = 0.209* | 99.13  ± 1.38 %  *p = 0.624* | 99.00  ± 2.25 %  *p = 0.024* |
|  | **2P vs 3P** | ***p = 0.268*** | ***p = 0.664*** | ***p = 0.360*** | ***p = 0.842*** | ***p = 0.927*** | ***p = 0.896*** | ***p = 0.780*** |  |
| **[^99m^Tc]Tc-exametazime** | 2P | 94.25 ± 2.02 %  *p = 0.036* | 93.25  ± 2.54 %  *p = 0.012* | 92.44  ± 3.77 %  *p = 0.001* | 90.5 ± 4.28 %  *p < 0.001* | 91.44 ± 4.40 %  *p < 0.001* | 89.75  ± 4.56 %  *p < 0.001* | 92.67  ± 4.69 %  *p = 0.001* | 86.00 ± 5.37 %  *p = 0.026* |
|  | 3P | 99.22 ± 0.55 %  *p = 0.487* | 97.63  ±0.63 %  *p = 0.052* | 99.00  ± 0.71 %  *p = 0.372* | 99.22 ± 0.66 %  *p = 0.487* | 97.63 ± 1.38 %  *p = 0.052* | 97.67 ± 1.03 %  *p = 0.037* | 96.78 ± 1.65 %  *p = 0.004* | 96.83 ± 0.31 %  *p < 0.001* |
|  | **2P vs 3P** | ***p = 0.105*** | ***p = 0.154*** | ***p = 0.010*** | ***p = 0.002*** | ***p = 0.014*** | ***p = 0.006*** | ***p = 0.105*** |  |
| **[^99m^Tc]Tc-oxidronate** | 2P | 100.11 ± 0.73 %  *p = 0.993* | 98.00  ± 0.53 %  *p = 0.016* | 97.90  ± 0.67 %  *p = 0.002* | 98.75 ± 0.86 %  *p = 0.052* | 99.64 ± 0.59 %  *p = 0.680* | 99.80  ± 0.57 %  *p = 0.551* | 99.18  ± 0.55 %  *p = 0.271* | 98.00  ± 1.00 %  *p = 0.451* |
|  | 3P | 99.45 ± 0.76 %  *p = 0.796* | 97.45  ± 1.45 %  *p = 0.115* | 98.18  ± 1.13 %  *p = 0.107* | 98.63 ± 1.26 %  *p = 0.189* | 98.36 ± 1.45 %  *p = 0.135* | 98.11  ± 1.78 %  *p = 0.176* | 96.44  ± 4.31 %  *p = 0.002* | 98.80  ± 0.58 %  *p < 0.001* |
|  | **2P vs 3P** | ***p = 0.824*** | ***p = 0.704*** | ***p = 0.784*** | ***p = 0.650*** | ***p = 0.228*** | ***p = 0.309*** | ***p = 0.014*** |  |
| **[^123^I]I-ioflupane** | 2P | 97.86  ± 0.26 %  *p = 0.016* | 96.11  ± 0.59 %  *p < 0.001* | 95.33  ± 1.08 %  *p < 0.001* | 99.13 ± 0.97 %  *p = 0.360* | 99.58 ± 0.87 %  *p = 0.704* | 100.33  ± 0.60 %  *p = 0.679* | 99.57  ± 0.43 %  *p = 0.639* | 96.83  ± 1.54 %  *p = 0.032* |
|  | 3P | 95.63  ± 1.81 %  *p = 0.001* | 94.11 ± 0.86 %  *p < 0.001* | 92.44  ± 1.80 %  *p < 0.001* | 95.78 ± 1.05 %  *p = 0.002* | 98.12 ± 1.39 %  *p = 0.171* | 97.13  ± 0.97 %  *p = 0.058* | 98.75  ± 0.96 %  *p = 0.475* | 98.33  ± 1.36 %  *p = 0.744* |
|  | **2P vs 3P** | ***p = 0.156*** | ***p = 0.207*** | ***p = 0.068*** | ***p = 0.033*** | ***p = 0. 343*** | ***p = 0.067*** | ***p = 0.713*** |  |
| **[^223^Ra]Cl_2_** | 2P | 97.67 ± 1.33 %  *p = 0.076* | 98.75  ± 1.31 %  *p = 0.557* | 100.89  ± 1.65 %  *p = 0.499* | 102.00  ± 2.06 %  *p = 0.071* | 105.00  ± 1.46 %  *p < 0.001* | 102.67  ± 1.81 %  *p = 0.043* | 103.89  ± 1.80 %  *p = 0.003* | 102.75 ± 2.17 %  *p = 0.339* |
|  | 3P | 100.89 ± 0.73 %  *p = 0.394* | 102.67  ± 0.99 %  *p = 0.011* | 103.13  ± 0.91 %  *p* *= 0.007* | 102.88  ± 1.11 %  *p = 0.015* | 103.00  ± 1.20 %  *p = 0.004* | 102.14  ± 0.80 %  *p = 0.042* | 107.11  ± 1.97 %  *p < 0.001* | 103.89  ± 1.62 %  *p = 0.683* |
|  | **2P vs 3P** | ***p = 0.055*** | ***p = 0.042*** | ***p = 0.245*** | ***p = 0.920*** | ***p = 0.234*** | ***p = 0.820*** | ***p = 0.055*** |  |
| **[^99m^Tc]Tc-besilesomab** | 2P | 101.33 ± 1.33 %  *p = 0.597* | 98.25  ± 1.35 %  *p = 0.365* | 99.33  ± 0.40 %  *p = 0.792* | 95.00  ± 2.68 %  *p = 0.047* | 91.11  ± 2.93 %  *p < 0.001* | 87.44  ± 4.34 %  p < 0.001 | 90.33  ± 4.07 %  *p < 0.001* | 90.20  ± 0.74 %  *p = 0.002* |
|  | 3P | 98.56 ± 0.90 %  *p = 0.718* | 98.44  ± 1.33 %  *p = 0.698* | 100.00  ± 0.75 %  *p = 0.934* | 87.56  ± 3.11 %  *p = 0.002* | 86.56  ± 6.44 %  *p = 0.001* | 93.57  ± 1.80 %  *p = 0.128* | 69.89  ± 6.90 %  *p < 0.001* | 74.83  ± 2.39 %  *p < 0.001* |
|  | **2P vs 3P** | ***p = 0.557*** | ***p = 0.870*** | ***p = 0.832*** | ***p = 0.115*** | ***p = 0.335*** | ***p = 0.218*** | ***p < 0.001*** |  |
| **[^99m^Tc]Tc-tetrofosmin** | 2P | 99.25 ± 0.41 %  *p = 0.567* | 100.56  ± 1.18 %  *p = 0.662* | 103.78  ± 1.42 %  *p = 0.003* | 101.89  ± 1.23 %  *p = 0.137* | 101.33  ± 0.99 %  *p = 0.348* | 100.25  ± 0.90 %  *p = 0.849* | 100.00  ± 0.90 %  *p = 1* | 102.80  ± 1.02 %  *p = 0.022* |
|  | 3P | 95.90 ± 2.17 %  *p = 0.331* | 93.50 ± 2.03 %  *p = 0.184* | 84.81 ± 6.09 %  *p < 0.001* | 79.90 ± 2.76 %  *p < 0.001* | 72.80 ± 2.94 %  *p < 0.001* | 68.36 ± 1.08 %  *p < 0.001* | 67.00 ± 2.58 %  *p < 0.001* | 59.71 ± 0.64 %  *p = 0.005* |
|  | **2P vs 3P** | ***p = 0.534*** | ***p = 0.184*** | ***p < 0.001*** | ***p < 0.001*** | ***p < 0.001*** | ***p < 0.001*** | ***p < 0.001*** |  |
| **[^99m^Tc]Tc-DMSA** | 2P | 99.44 ± 1.37 %  *p = 0.689* | 98.00  ± 0.73 %  *p = 0.163* | 99.11  ± 0.98 %  *p = 0.522* | 97.00  ± 0.93 %  *p = 0.036* | 95.13  ± 1.61 %  *p = 0.001* | 96.37  ± 1.00 %  *p = 0.011* | 94.50  ± 1.34 %  *p < 0.001* | 94.00 ± 1.63 %  *p = 0.197* |
|  | 3P | 113.38 ± 4.90 %  p < 0.001 | 104.75  ± 2.43 %  *p = 0.310* | 106.71  ± 2.64 %  *p* *= 0.069* | 91.25  ± 2.49 %  *p = 0.014* | 82.06  ± 3.22 %  *p < 0.001* | 78.33  ± 3.52 %  *p < 0.001* | 70.84  ± 4.07 %  *p < 0.001* | 62.56  ± 0.87 %  *p = 0.683* |
|  | **2P vs 3P** | ***p* = 0.002** | ***p = 0.218*** | ***p = 0.112*** | ***p = 0.188*** | ***p = 0.007*** | ***p < 0.001*** | ***p < 0.001*** |  |
| **[^99m^Tc]Tc-nanocolloids** | 2P | 100.63 ± 0.93 %  *p = 0.495* | 101.00  ± 0.55 %  *p = 0.198* | 100.22  ± 0.76 %  *p = 0.775* | 100.00  ± 0.63 %  *p = 0.939* | 99.50  ± 0.68 %  *p = 0.471* | 100.25  ± 0.75 %  *p = 0.698* | 99.00  ± 0.44 %  *p = 0.221* | 103.00 ± 3.00 %  *p = 0.003* |
|  | 3P | 94.55 ± 1.29 %  *p = 0.032* | 92.50 ± 1.20 %  *p = 0.010* | 89.50 ± 1.73 %  *p < 0.001* | 89.50 ± 3.43 %  *p < 0.001* | 85.33 ± 3.25 %  *p < 0.001* | 79.89 ± 6.49 %  *p < 0.001* | 80.50 ± 4.98 %  *p < 0.001* | 86.00 ± 3.75 %  *p = 0.880* |
|  | **2P vs 3P** | ***p = 0.046*** | ***p = 0.012*** | ***p = 0.001*** | ***p < 0.001*** | ***p < 0.001*** | ***p < 0.001*** | ***p < 0.001*** |  |
